# Supplementary material for: Assessing the living and dead proportions of cold-water coral colonies: implications for deep-water Marine Protected Area monitoring in a changing ocean
Source: PeerJ. 2017 Oct 5;5:e3705. doi: 10.7717/peerj.3705 (PMC5632539; doi:10.7717/peerj.3705)
Supplement: Supplemental Information 1 [file peerj-05-3705-s001.pdf]

Site	Dive	Coral	layer thickness	Mean	SD	Living layer thickness	Mean	SD	Whole colony size	Mean	SD
			675			92			767.00		
			807			125			932.00		
			823			171			994.00		
			731			106			837.00		
	1	c1	697	747	66	137	126	30	834.00	873	90
			250			42			292.00		
			250			41			291.00		
			319			53			372.00		
			301			68			369.00		
Mingulay	3	c1	259	276	32	35	48	13	294.00	324	43
			425			128			553.00		
			526			138			664.00		
			544			144			688.00		
			545			141			686.00		
	3	c2	448	498	57	129	136	7	577.00	634	64
			725			184			909.00		
			687			158			845.00		
			655			183			838.00		
			701			195			896.00		
	5	c1	693	692	25	161	176	16	854.00	868	32
			605			261			866.00		
			716			246			962.00		
Banana			731			273			1004.00		
			814			257			1071.00		
	7	c1	728	719	75	262	260	10	990.00	979	75
			611			64			675.00		
			583			71			654.00		
			567			92			659.00		
			561			66			627.00		
	8	c1	599	584	21	88	76	13	687.00	660	23
			372			156			528.00		
			472			130			602.00		
			492			108			600.00		
			499			114			613.00		
	8	c2	482	463	52	136	129	19	618.00	592	37
			912			195			1107.00		
			1030			171			1201.00		
			1051			227			1278.00		
			1067			237			1304.00		
	8	c3	924	997	73	207	207	26	1131.00	1204	87
			857			103			960.00		

			1046			109			1155.00		
			871			113			984.00		
			965			105			1070.00		
	8	c4	963	940	78	121	110	7	1084.00	1051	79
			1061			223			1284.00		
			966			186			1152.00		
Mingulay			1081			220			1301.00		
			1110			190			1300.00		
	8	c5	1008	1045	58	201	204	17	1209.00	1249	66
			616			137			753.00		
			677			136			813.00		
			560			171			731.00		
			744			154			898.00		
	8	c6	777	675	89	203	160	28	980.00	835	104
			1186			194			1380.00		
			934			192			1126.00		
			1065			168			1233.00		
			1110			200			1310.00		
	10	c1	1053	1070	92	224	196	20	1277.00	1265	95
			1303			134			1437.00		
			1117			120			1237.00		
			1035			167			1202.00		
			1284			134			1418.00		
	10	c2	1300	1208	124	127	136	18	1427.00	1344	115
			269			79			348.00		
			259			70			329.00		
			282			92			374.00		
			262			65			327.00		
	41	c1	268	268	9	74	76	10	342.00	344	19
			1281			188			1469.00		
			854			103			957.00		
			798			127			925.00		
			1043			155			1198.00		
	31	c1	846	964	200	166	148	33	1012.00	1112	226
			750			180			930.00		
			767			154			921.00		
			778			147			925.00		
			913			152			1065.00		
Pisces	31	c2	691	780	82	191	165	19	882.00	945	70
			1120			231			1351.00		
			892			158			1050.00		
			877			145			1022.00		
	32	c1	894	946	116	191	181	38	1085.00	1127	152

			817			183			1000.00		
			906			217			1123.00		
			847			171			1018.00		
			764			199			963.00		
	32	c2	806	828	53	132	180	32	938.00	1008	71
