# Supplementary figures and images for: Assessing the living and dead proportions of cold-water coral colonies: implications for deep-water Marine Protected Area monitoring in a changing ocean
Source: PeerJ. 2017 Oct 5;5:e3705. doi: 10.7717/peerj.3705 (PMC5632539; doi:10.7717/peerj.3705)

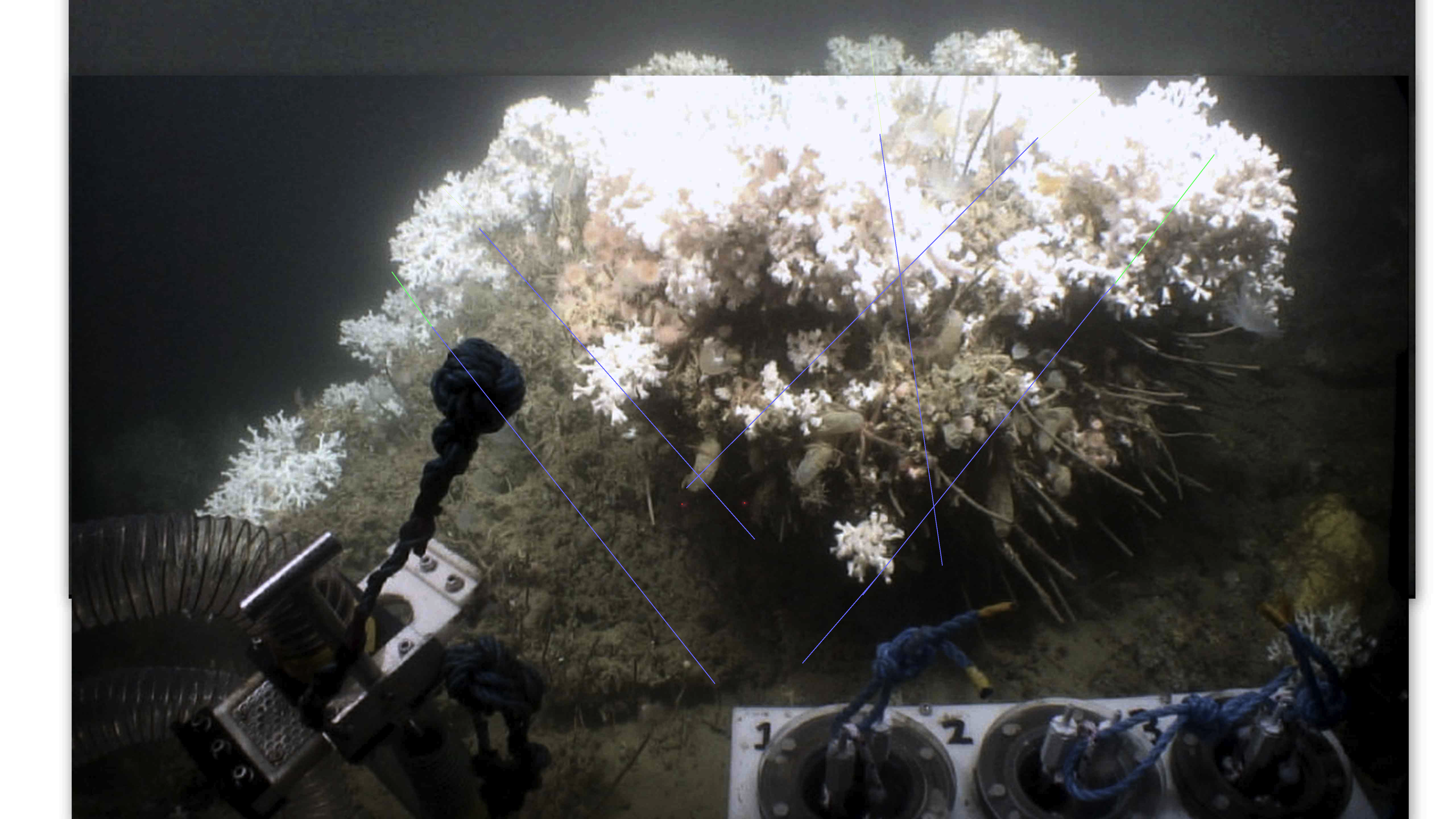

Supplement: Supplemental Information 2 [file peerj-05-3705-s002.png]

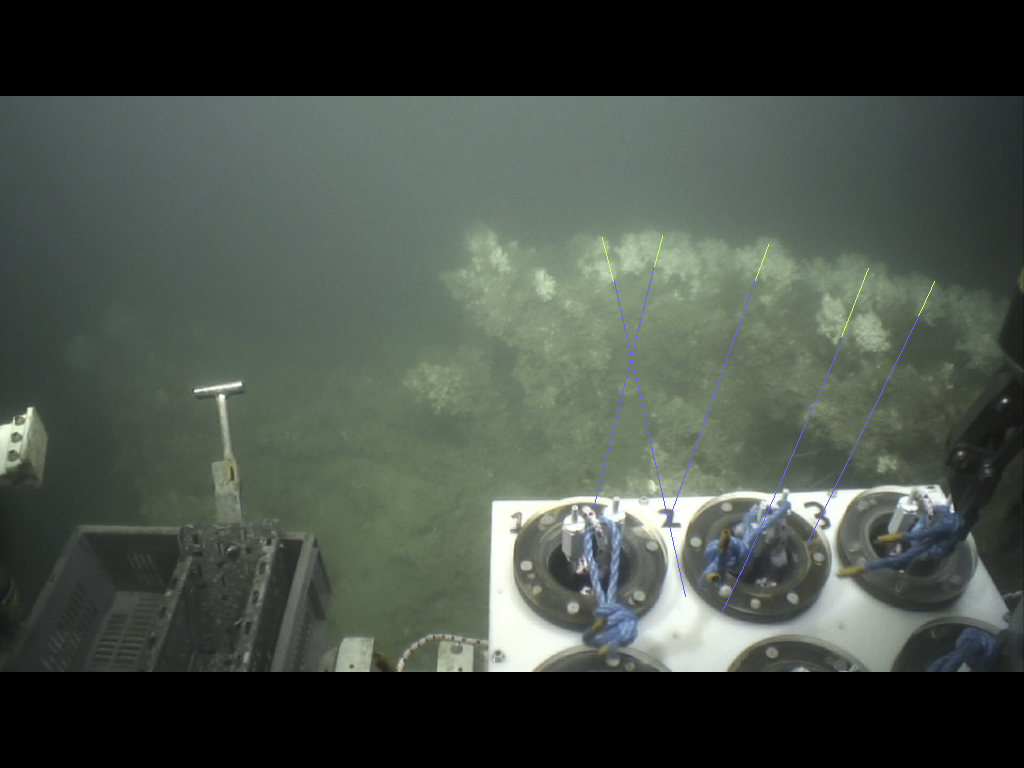

Supplement: Supplemental Information 3 [file peerj-05-3705-s003.png]

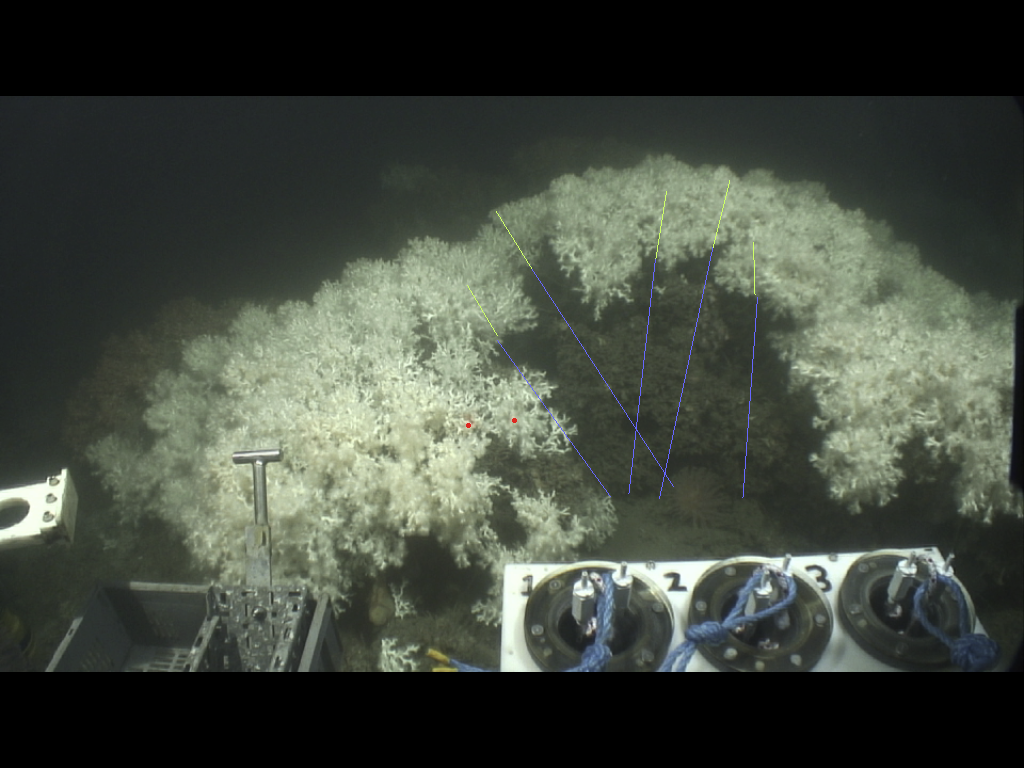

Supplement: Supplemental Information 4 [file peerj-05-3705-s004.png]

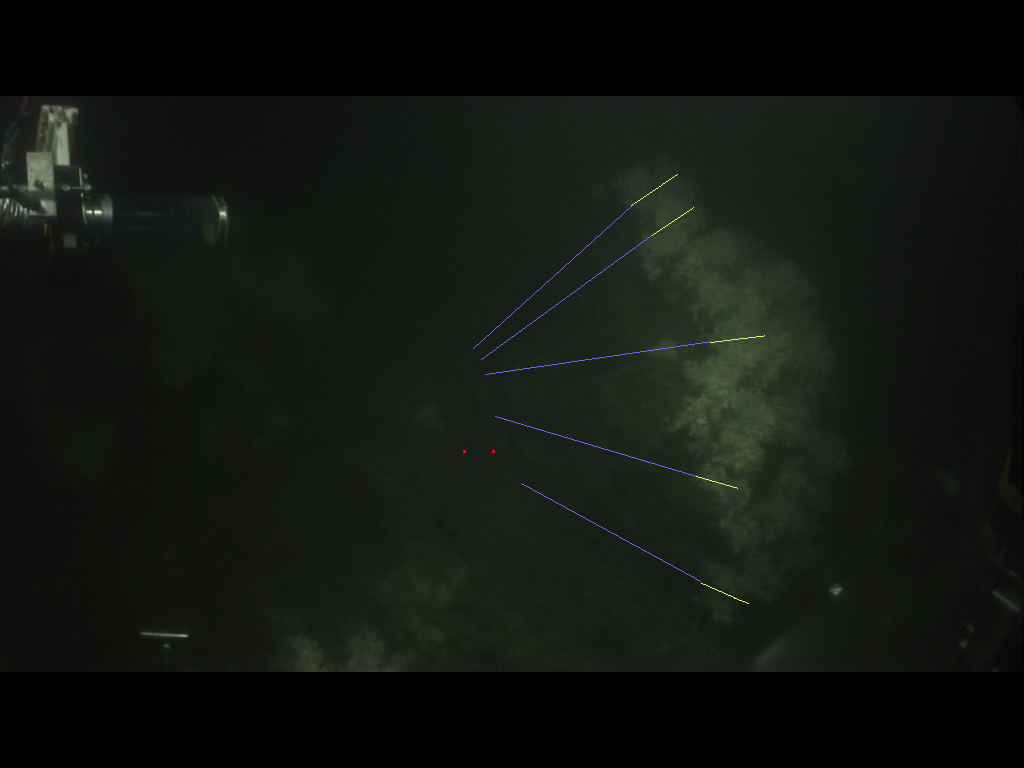

Supplement: Supplemental Information 5 [file peerj-05-3705-s005.png]

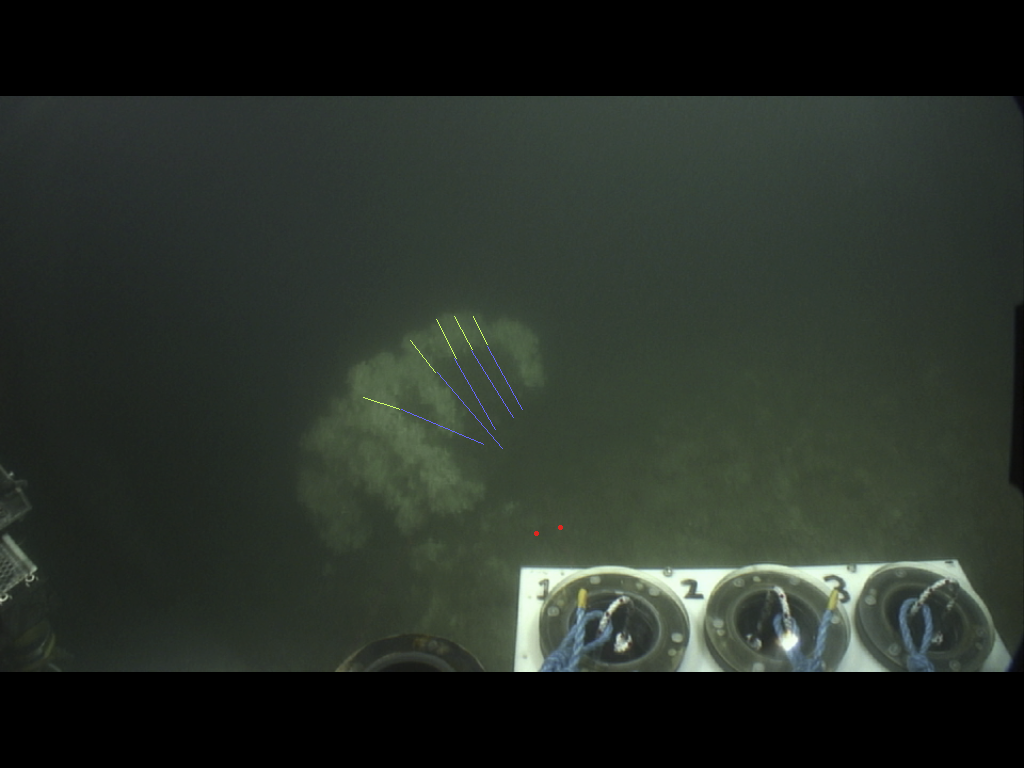

Supplement: Supplemental Information 6 [file peerj-05-3705-s006.png]

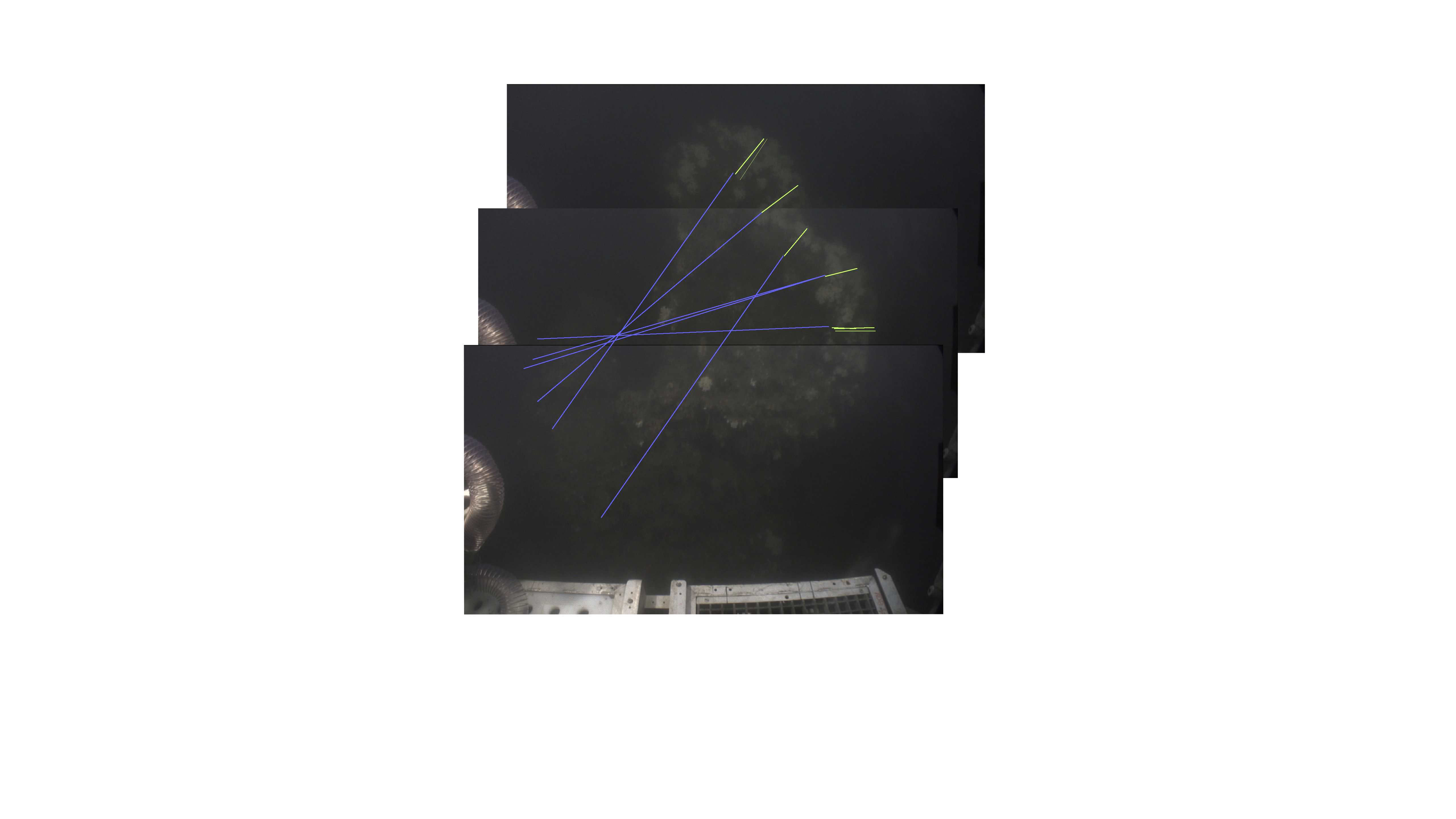

Supplement: Supplemental Information 7 [file peerj-05-3705-s007.png]

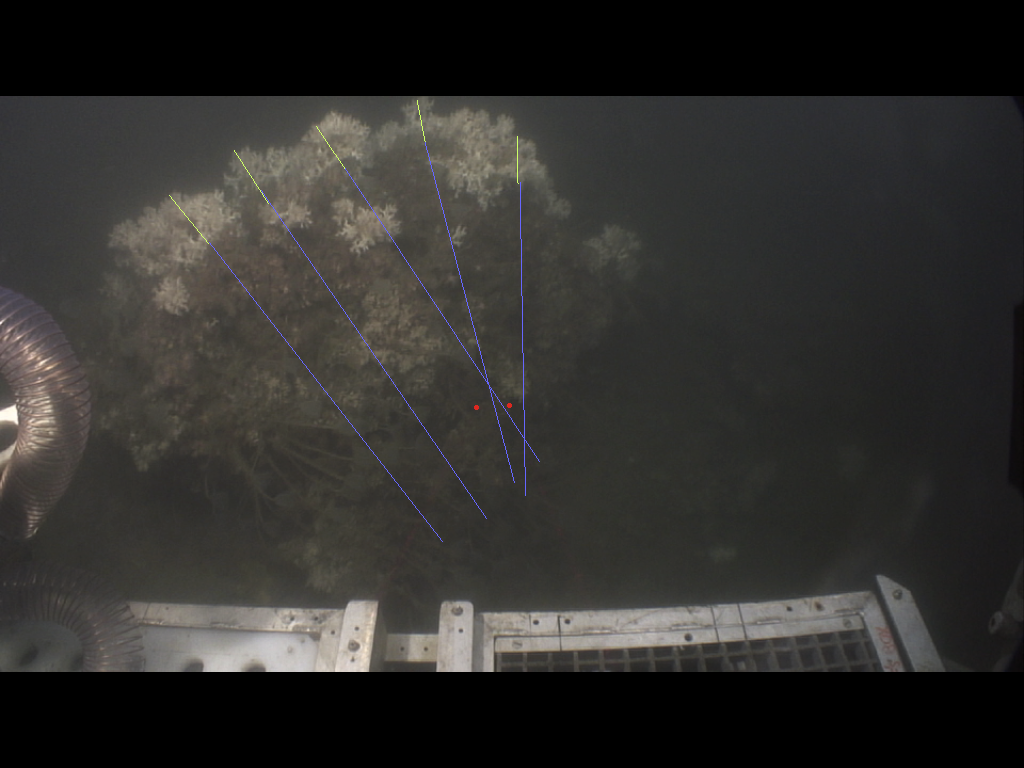

Supplement: Supplemental Information 8 [file peerj-05-3705-s008.png]

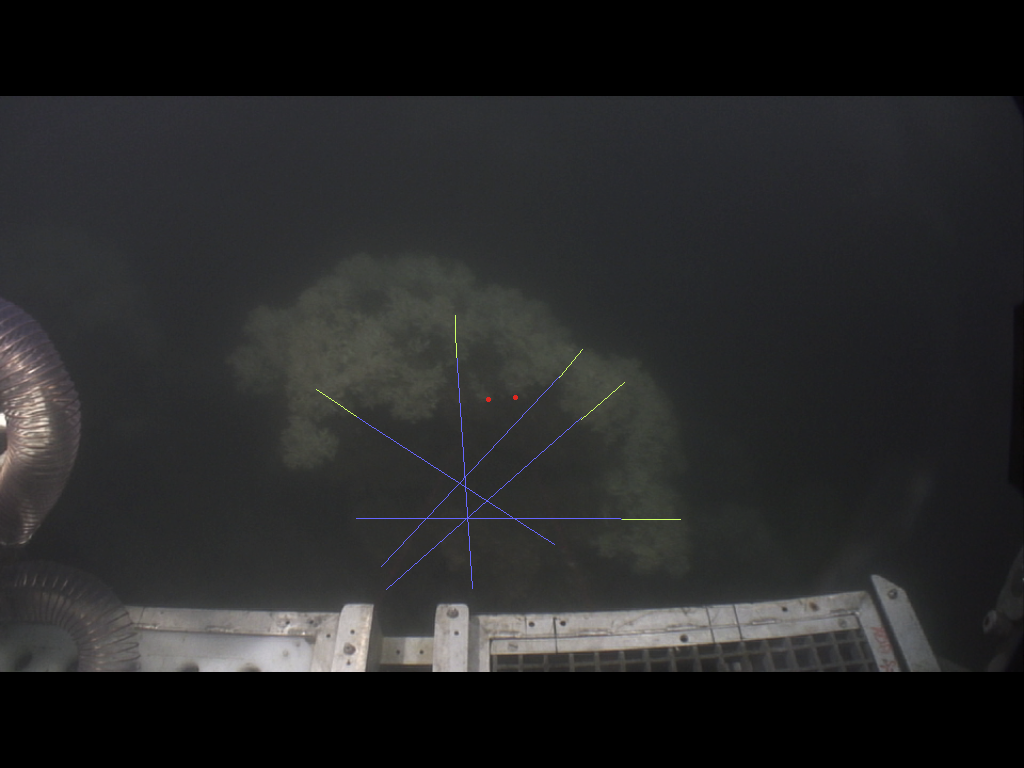

Supplement: Supplemental Information 9 [file peerj-05-3705-s009.png]

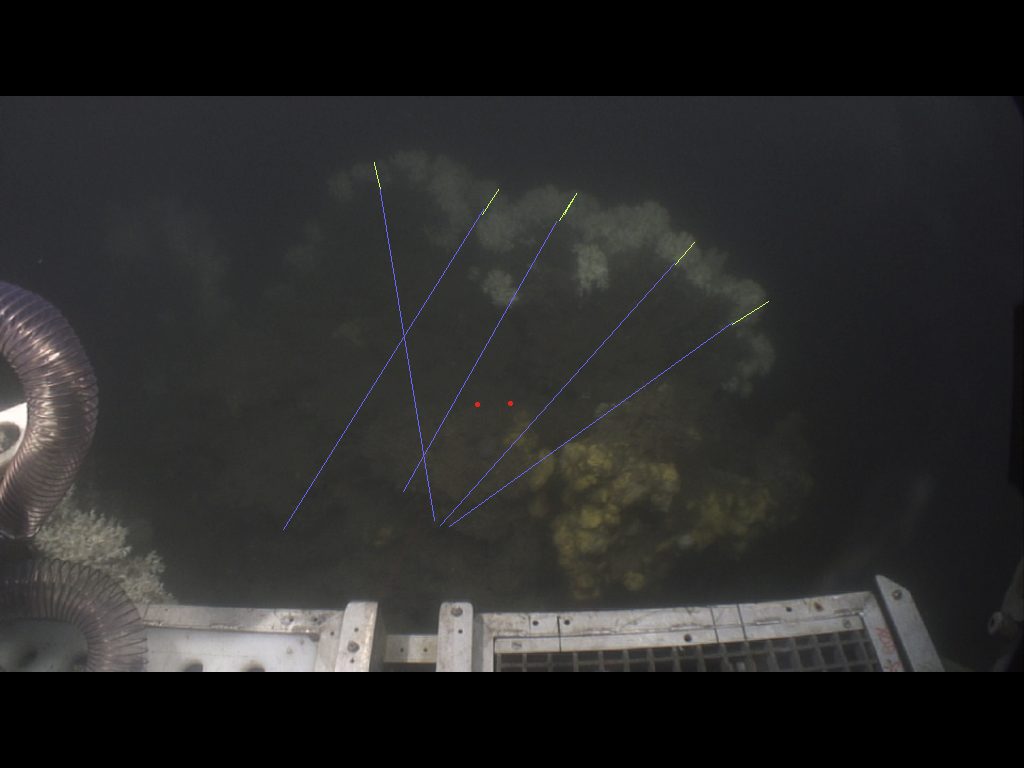

Supplement: Supplemental Information 10 [file peerj-05-3705-s010.png]

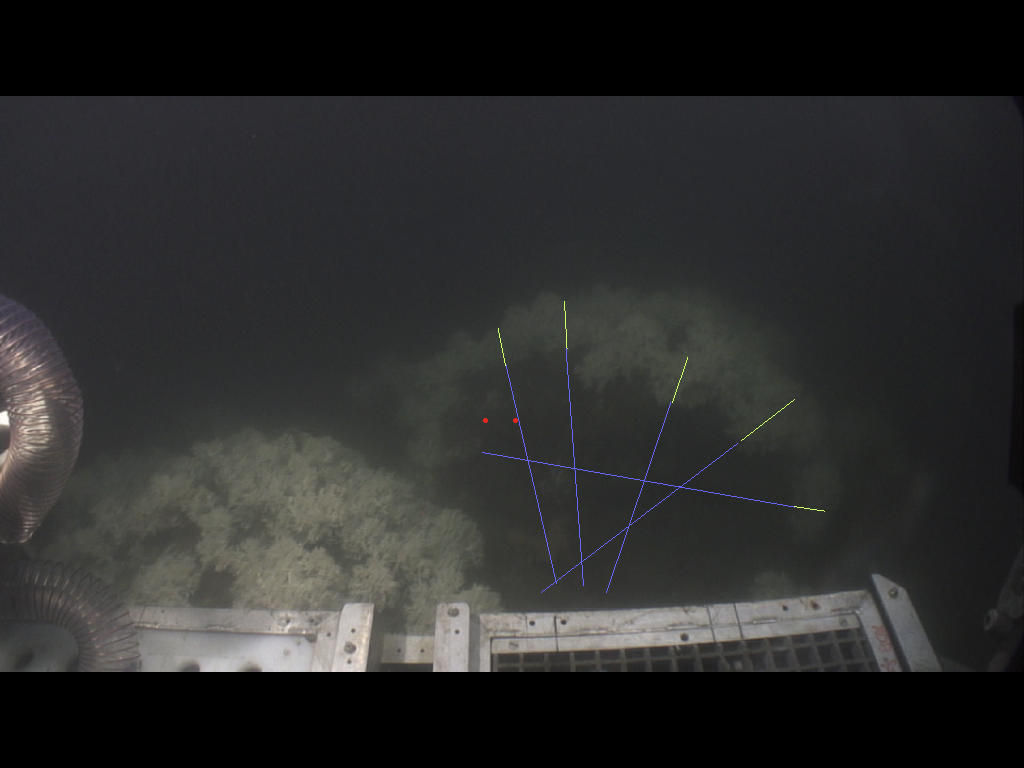

Supplement: Supplemental Information 11 [file peerj-05-3705-s011.png]

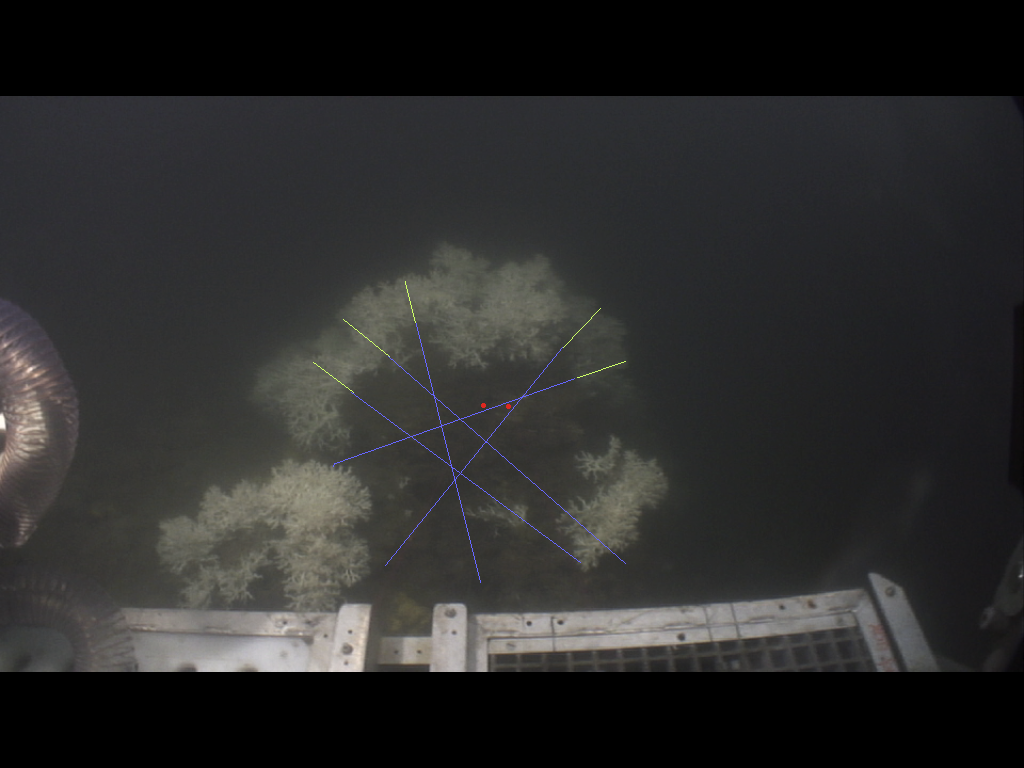

Supplement: Supplemental Information 12 [file peerj-05-3705-s012.png]

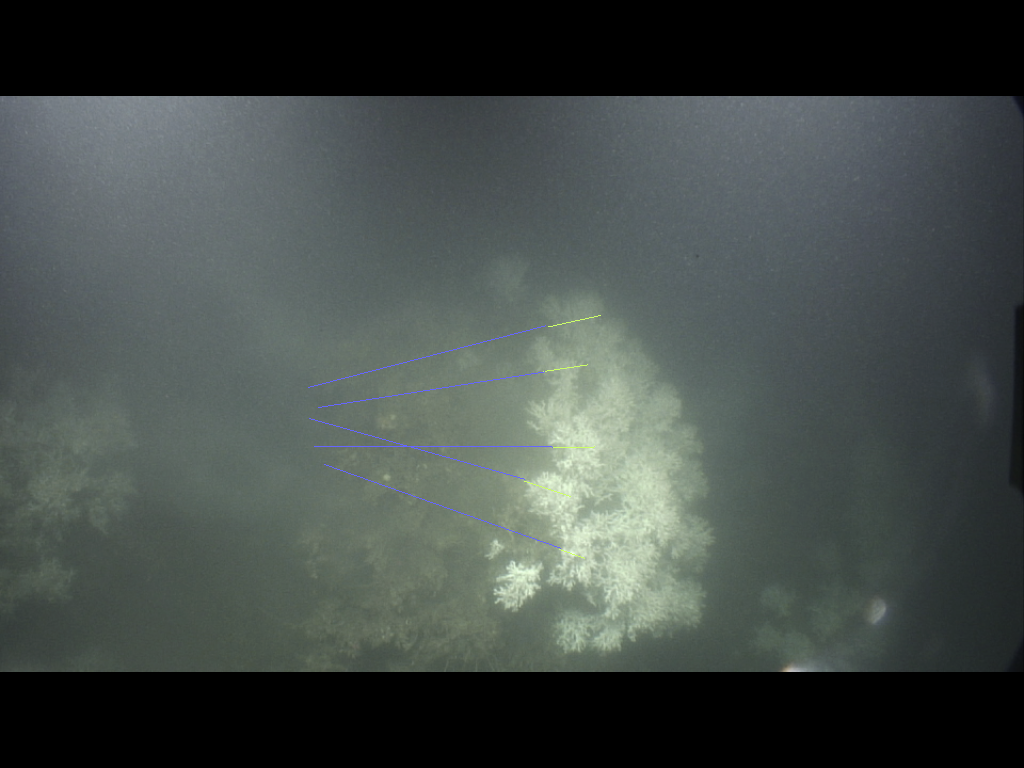

Supplement: Supplemental Information 13 [file peerj-05-3705-s013.png]

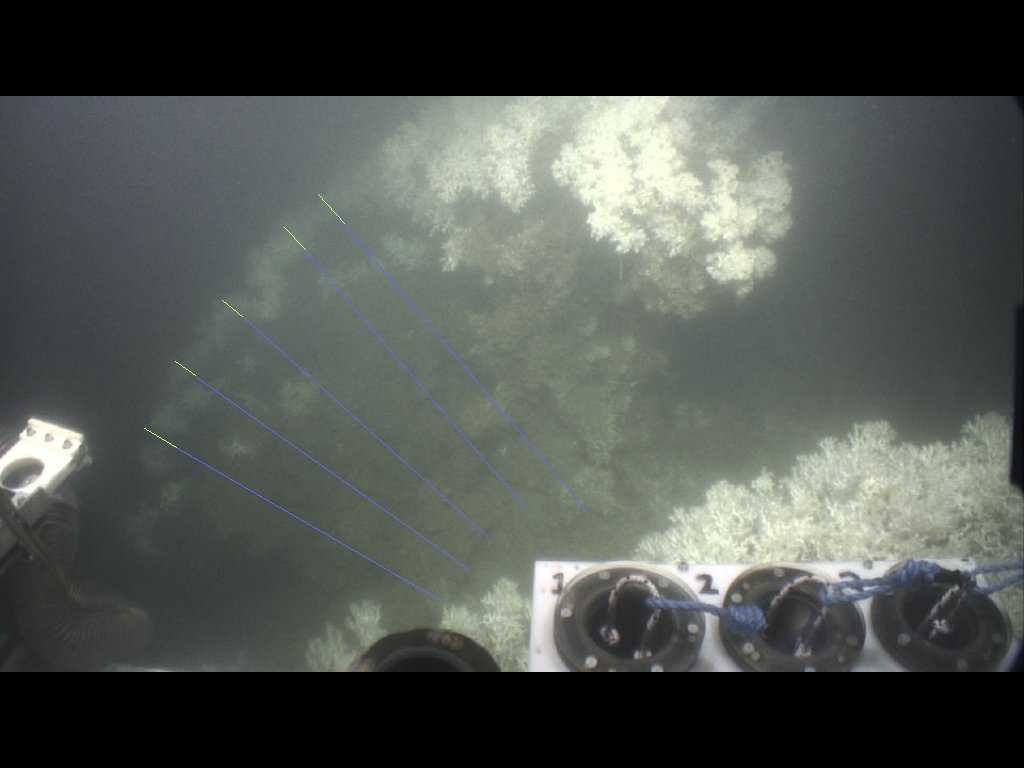

Supplement: Supplemental Information 14 [file peerj-05-3705-s014.png]

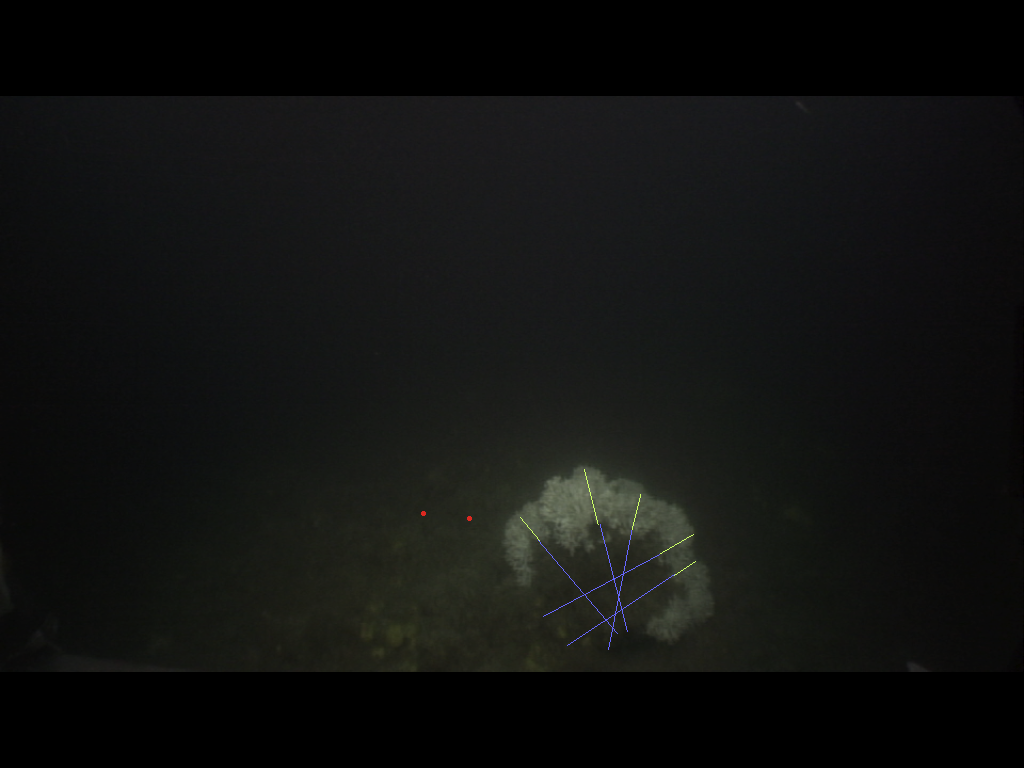

Supplement: Supplemental Information 15 [file peerj-05-3705-s015.png]

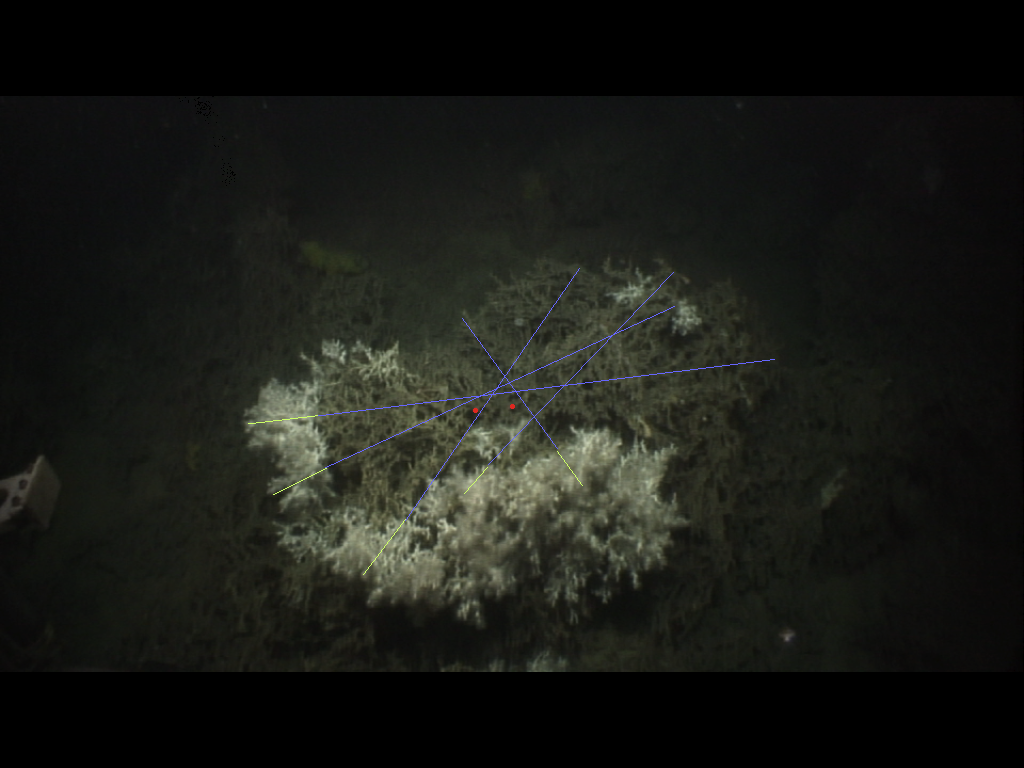

Supplement: Supplemental Information 16 [file peerj-05-3705-s016.png]

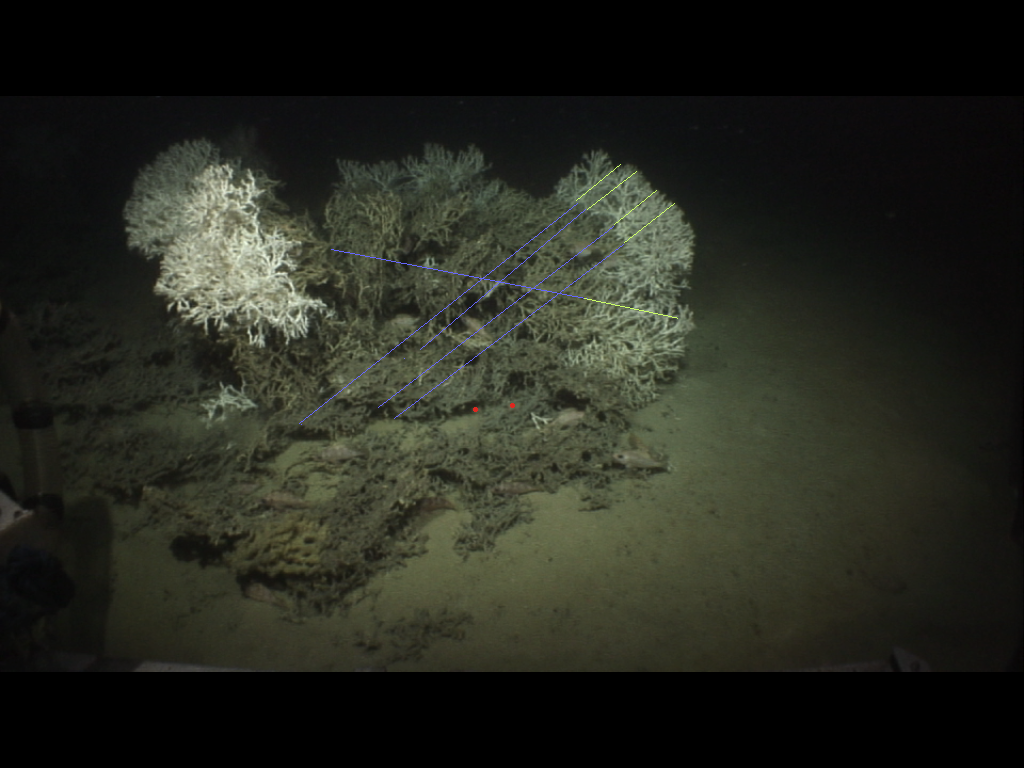

Supplement: Supplemental Information 17 [file peerj-05-3705-s017.png]

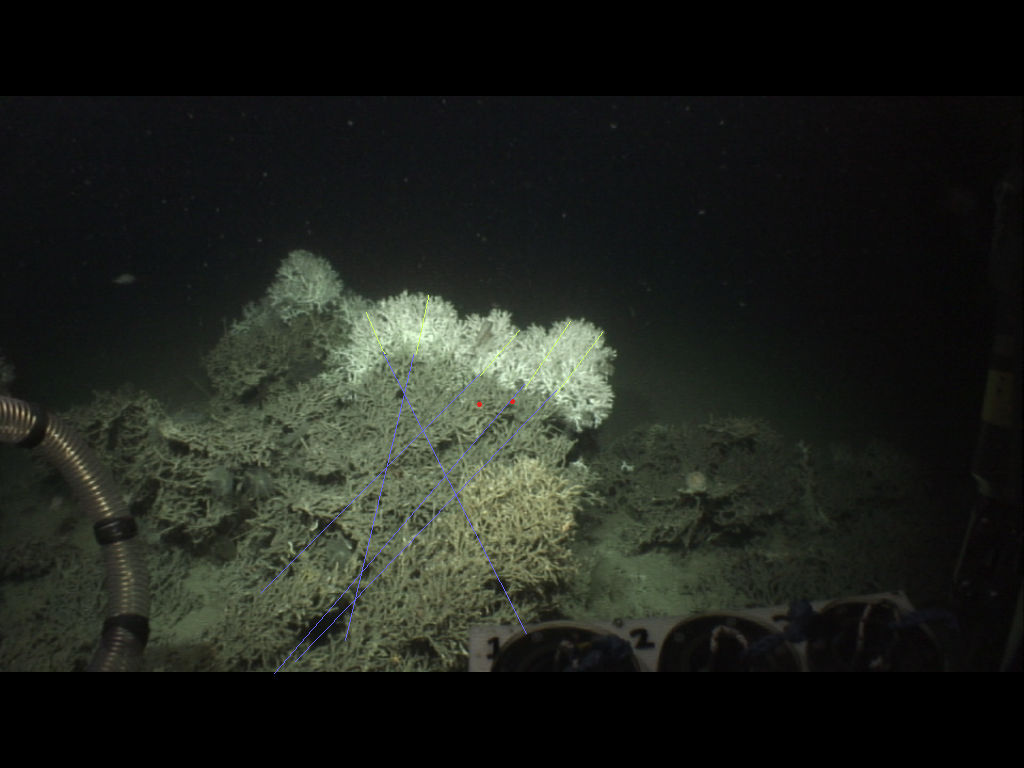

Supplement: Supplemental Information 18 [file peerj-05-3705-s018.png]

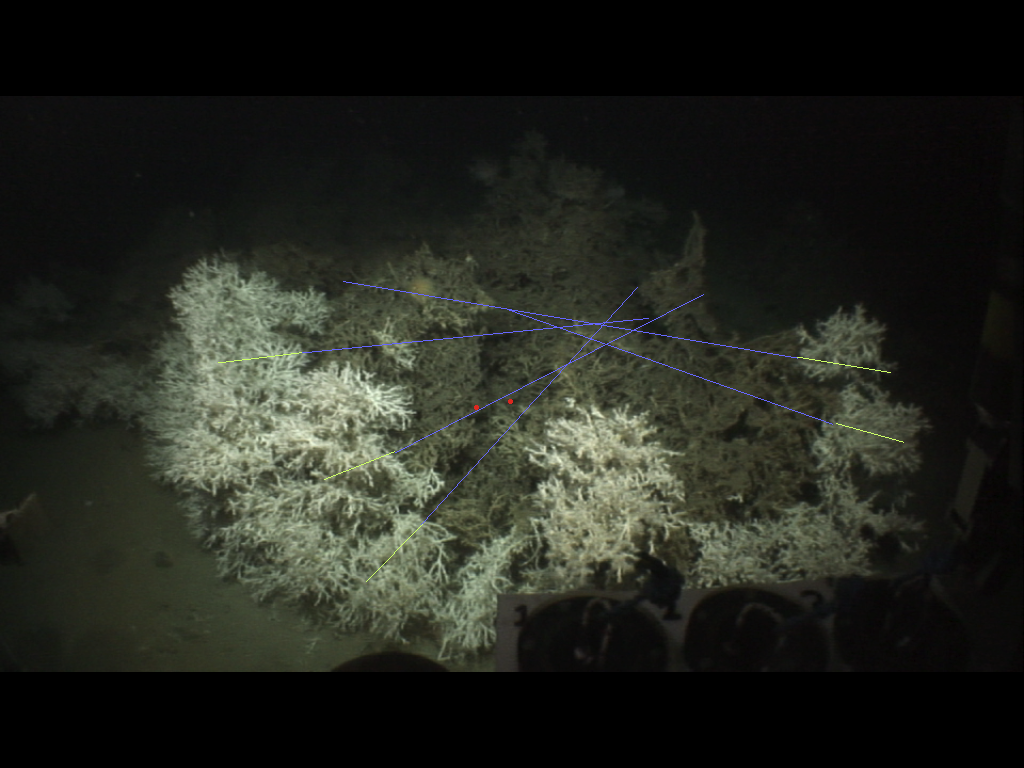

Supplement: Supplemental Information 19 [file peerj-05-3705-s019.png]
